# Colon Microbiome of Pigs Fed Diet Contaminated with Commercial Purified Deoxynivalenol and Zearalenone

**DOI:** 10.3390/toxins10090347

**Published:** 2018-08-29

**Authors:** Kondreddy Eswar Reddy, Jin Young Jeong, Jaeyong Song, Yookyung Lee, Hyun-Jeong Lee, Dong-Wook Kim, Hyun Jung Jung, Ki Hyun Kim, Minji Kim, Young Kyoon Oh, Sung Dae Lee, Minseok Kim

**Affiliations:** 1 Animal Nutrition & Physiology Team, National Institute of Animal Science, Rural Development Administration, #1500 Kongjwipatjwi-ro, Iseo-myeon, Wanju 55365, Korea; dreswar4u@gmail.com (K.E.R.); jeong73@korea.kr (J.Y.J.); jysong76@korea.kr (J.S.); yoo3930@korea.kr (Y.L.); hyunj68@korea.kr (H.-J.L.); poultry98@korea.kr (D.-W.K.); hyjjung@korea.kr (H.J.J.); kihyun@korea.kr (K.H.K.); mjkimen@naver.com (M.K.); oh665@korea.kr (Y.K.O.); 2 Department of Poultry Science, Korea National College of Agriculture and Fisheries, #1515 Kongjwipatjwi-ro, Deokjin-gu, Jeonju-si 54874, Korea; 3 Department of Animal Science, College of Agriculture and Life Sciences, Chonnam National University, Gwangju 61186, Korea

**Keywords:** deoxynivalenol, zearalenone, pig, colon microbiota, *Lactobacillus*, detoxification

## Abstract

Deoxynivalenol (DON) and zearalenone (ZEN) can seriously affect animal health, with potentially severe economic losses. Previous studies have demonstrated that gut microbiota plays a significant role in detoxification. We analyzed the colon contents from three groups of pigs (fed either a standard diet, or a diet with 8 mg/kg DON or ZEN). Bacterial 16S rRNA gene amplicons were obtained from the colon contents, and sequenced using next-generation sequencing on the MiSeq platform. Overall, 2,444,635 gene sequences were generated, with ≥2000 sequences examined. Firmicutes and Bacteroidetes were the dominant phyla in all three groups. The sequences of *Lactobacillus*, *Megasphaera*, and *Faecalibacterium* genera, and the unclassified Clostridiaceae family, represented more than 1.2% of the total, with significantly different abundances among the groups. *Lactobacillus* was especially more abundant in the DON (7.6%) and ZEN (2.7%) groups than in the control (0.2%). A total of 48,346 operational taxonomic units (OTUs) were identified in the three groups. Two OTUs, classified as *Lactobacillus*, were the most dominant in the DON and ZEN groups. The abundances of the remaining OTUs were also significantly different among the groups. Thus, the mycotoxin-contaminated feed significantly affected the colon microbiota, especially *Lactobacillus*, which was the most abundant. Therefore, we speculate that *Lactobacillus* plays a major role in detoxification of these mycotoxins.

## 1. Introduction

Deoxynivalenol (DON) and zearalenone (ZEN) are *Fusarium* mycotoxins that cause significant economic losses of crops globally, and frequently contaminate food and animal feed, such as maize, wheat, barley, rice, rye, oats, sorghum, and triticale. DON and ZEN cause damage to the gastrointestinal and immune systems in both humans and farm animals, resulting in vomiting, diarrhea, hemorrhage, leukopenia, and shock [1,2,3]. Among animal species, pigs show a comparatively high sensitivity to DON and ZEN, likely because of a high percentage of cereals in the porcine diet, posing a greater risk of exposure to these two mycotoxins. Furthermore, the front portion of the pig small intestine lacks microorganisms that are able to degrade mycotoxins before they are absorbed by the small intestine [4,5].

DON has been known to cause toxic effects in both animals and humans. Following DON exposure, the initial adverse effect in pigs is a reduced feed intake. DON adversely affects the growth performance, immune response, and reproductive performance in growing pigs [6,7]. A DON-contaminated diet influences the gastrointestinal tract, causing epithelial wounds in the stomach and intestine and, foremost, an intestinal inflammatory response in pigs [8,9]. According to Pierron et al. [10], in vitro and in vivo studies confirmed that DON severely inhibited intestinal nutrient absorption, changed the intestinal cell functions, and caused severe intestinal barrier damage.

ZEN and its metabolites clinically cause hyperestrogenism and reproductive disorders. ZEN metabolites compete with endogenous estrogens for estrogen receptor binding sites, and ultimately affect RNA and protein synthesis, leading to the deregulation of estrogenic activities [11]. Compared to chickens and ruminants, pigs are more sensitive to ZEN exposure, even at low levels [12]. According to Dänicke et al. [13], ZEN is regularly found in piglets under normal conditions, and these animals are initially exposed to ZEN in the uterus through placental transfer from the exposed sow, or through release of stored ZEN via suckling. ZEN also shows numerous genotoxic and cytotoxic effects in vitro and ex vivo, and is potentially carcinogenic [14]. Further, ZEN is recognized to be immunotoxic; nonetheless, its function in the inflammatory response is not yet completely understood [15].

The animal gastrointestinal tract is colonized by a rich variety of microorganisms, and quantitative and qualitative permanence of these organisms is an important factor in the maintenance of animal health [16]. The gut microbiota exerts obvious effects on basic host physiology and metabolism [17]. According to Richards et al. [16], the animal intestinal microbiota exhibits nutritional and protective functions, stimulates the immunity, produces fermentation products, and prevents colonization by pathogens. Bauer and his team [18] have demonstrated that the gut microbiota is influenced by various factors, such as the animal health, individual characteristics of the animal, and the feed type and quality. Microorganisms may also prevent harmful effects on animal health; in fact, it has been suggested that intestinal microbiota may play a protective role against mycotoxins as potential risk factors for inflammatory bowel disease [19]. As shown by Bauer and Williams [18], pathogenic microorganisms produce toxic metabolites and fecal enzymes that may increase the levels of carcinogenic substances, or convert procarcinogenic compounds into carcinogenic ones.

However, studies of the effects of the DON and ZEN mycotoxins on the pig gastrointestinal microbiota are lacking; in particular, the effect of ZEN on the pig gut microbiota remains unclear. Recently, Li et al. [20] have studied the intestinal microbial diversity in pigs fed a basal diet, naturally DON-contaminated wheat, and a feed contaminated with *Clostridium* sp. WJ06. In another study, DON and ZEN, separately and together, induced negative effects on the microbial diversity in the ascending colon of pigs [21].

Recently, 16S rRNA gene (*rrs*) sequencing using the Illumina MiSeq platform was used to compare the intestinal bacterial biota in DON-contaminated wheat-fed pigs and the rumen microbiota of steers fed various diets, demonstrating that DON and different diets greatly affected the microbiota of pigs and cattle, respectively [20,22]. However, direct effects of purified DON and ZEN on the colon microbiota composition have not yet been reported in pigs. Therefore, the main aim of this study was to comparatively investigate the abundance of unculturable microorganisms in control and mycotoxin-treated groups of pigs to gain insights into the community structure of the pig colon microbiota. We hypothesized that the community structure of the colon microbiome of pigs would be influenced by toxic effects of DON and ZEN.

## 2. Results

### 2.1. Sequencing and Bacterial Abundance

Quality control of clean *rrs* sequences, obtained from the colon contents of the control, DON, and ZEN dietary groups, resulted in a total of 2,444,635 sequences, with read lengths averaging 500 nucleotides. Of these, 740,744 sequences represented control feces, while 884,437 and 819,454 represented DON and ZEN feces (Table 1). The numbers of *rrs* sequences from individual control samples ranged from 158,817 to 192,996; those from DON-treated samples ranged from 166,967 to 196,160, while those from ZEN-treated samples ranged from 140,276 to 195,822.

**Figure 1:**
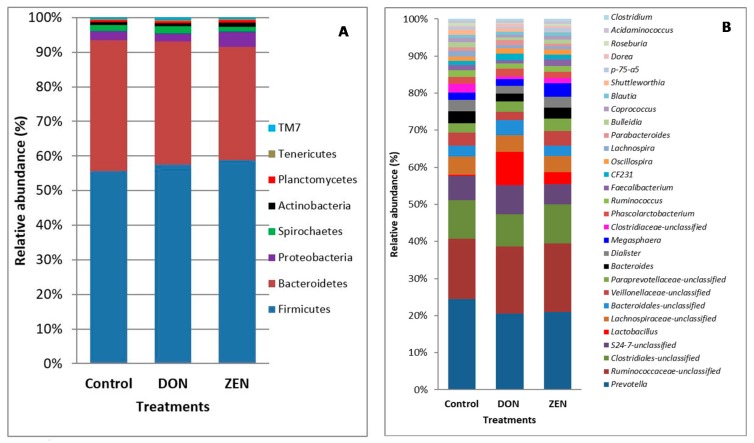
Microbial taxonomic profiles from the colon contents of the three dietary treatment groups at the phylum (**A**) and genus (**B**) levels, classified by the representation of >1% of the total sequences. Taxonomic compositions of the colon microbiota among the control, deoxynivalenol (DON), and zearalenone (ZEN) groups were compared based on the relative abundance (taxon reads/total reads in the colon contents).

Almost 99.8% of all *rrs* sequences from the colon contents of the three groups were classified into known phyla. As shown in Figure 1 and Supplementary Table S1, the phyla Firmicutes and Bacteroidetes were highly abundant, and were represented by 57.3% and 35.00% of all *rrs* sequences, respectively. The next most abundant phylum was Proteobacteria, which was represented by 3.2% of the total sequences; the remaining phyla were each represented by <2.00% of all *rrs* sequences. The phyla Spirochaetes and Actinobacteria were represented by 1.7% and 1.0% of all *rrs* sequences, respectively. Planctomycetes, Tenericutes, and candidate division TM7 were represented by less than 1.00% of all *rrs* sequences. The genus *Prevotella* comprised 23.7% of the 5,279,245 *rrs* sequences.

At the genus level, *Prevotella* was highly abundant, being represented by 18.8% of the 2,444,635 *rrs* sequences. Other genera, represented by at least 0.5% of the total sequences, were *Lactobacillus* (3.8%), *Dialister* (2.3%), *Bacteroides* (2.3%), *Megasphaera* (2.1%), *Phascolarctobacterium* (1.6%), *Treponema* (1.6%), *Ruminococcus* (1.4%), *Faecalibacterium* (1.2%), CF231 (1.2%), *Oscillospira* (1.1%), *Lachnospira* (1.0%), *Bulleidia* (0.9%), *Coprococcus* (0.8%), *Parabacteroides* (0.8%), *Blautia* (0.8%), *Campylobacter* (0.8%), *Shuttleworthia* (0.7%), p-75-a5 (0.5%), *Dorea* (0.5%), *Roseburia* (0.5%), *Acidaminococcus* (0.5%), *Clostridium* (0.5%), and *Desulfovibrio* (0.5%). Some taxa were not classified at the genus level but showed high abundance at the phylum level, such as Firmicutes and Bacteroidetes. The orders Clostridiales (8.5%) and Bacteroidales (2.8%) were dominant, and at the family level, Ruminococcaceae (14.4%) was highly abundant, followed by S24-7 (5.7%), Lachnospiraceae (3.9%), Veillonellaceae (2.7%), Paraprevotellaceae (2.5%), Clostridiaceae (1.3%), Christensenellaceae (0.5%), and Erysipelotrichaceae (0.5%).

A total of 192,724 operational taxonomic units (OTUs) were identified at a 0.03 dissimilarity cutoff level for all samples. All reads were normalized and analyzed using the Shannon diversity index, Chao1 richness estimator, and the Simpson index. OTUs of normalized samples did not vary (*p* > 0.05) among the three dietary treatment groups. The data of the richness estimate showed significant differences (*p* < 0.05) in the numbers of OTUs among the groups (Table 1).

### 2.2. Taxonomic Composition

Analysis of the taxonomic abundance in samples from the control, DON, and ZEN dietary groups showed differences at the phylum and genus levels (Figure 1A,B and Supplementary Table S1). The microbial data for all three dietary treatment groups were analyzed to determine the mean comparative abundance (taxon reads/total reads in a sample). At the genus level, *Lactobacillus* (*p* = 0.002), *Megasphaera* (*p* = 0.01), and *Faecalibacterium* (*p* = 0.045), as well as the order Clostridiales (*p* = 0.05), showed significant differences in the abundance among the DON, ZEN, and control groups. Analysis of the abundances at the taxonomic levels from the phylum to genus revealed differences among the DON, ZEN, and control groups. These variations were most obvious for Firmicutes, Bacteroidetes, *Prevotella*, *Lactobacillus*, the family Ruminococcaceae, and the order Clostridiales.

**Figure 2:**
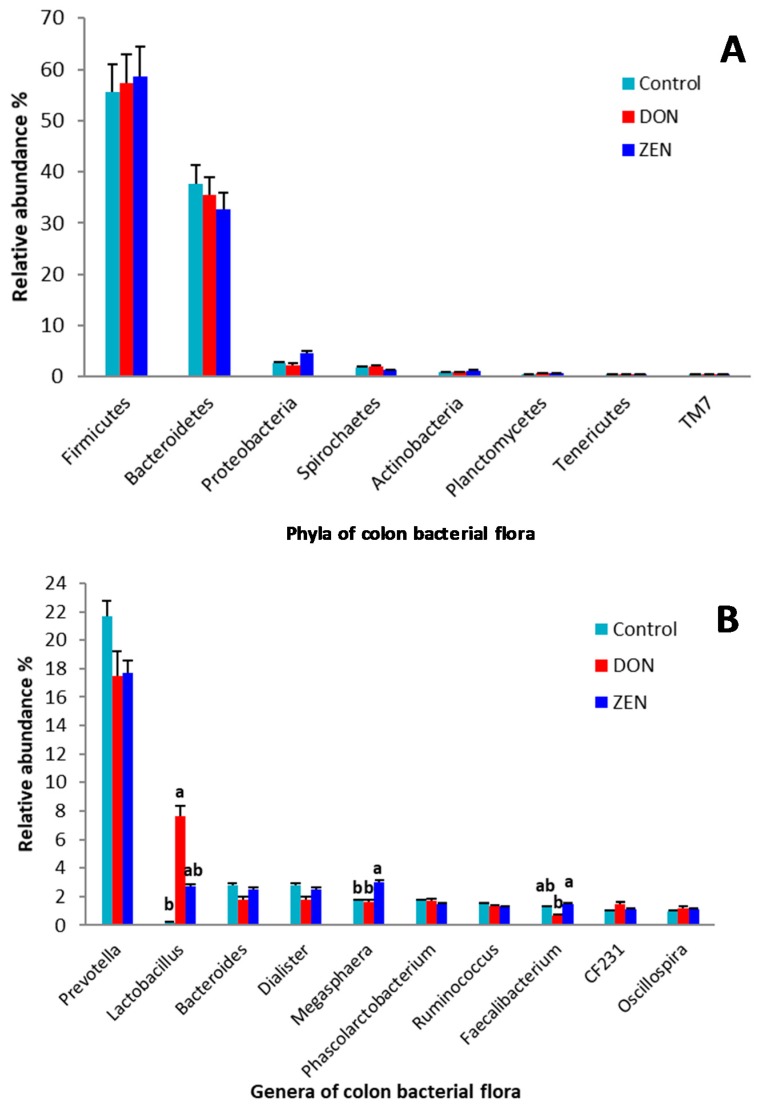
Relative abundances of the colon microbiota between the control and deoxynivalenol (DON) and zearalenone (ZEN) mycotoxin groups. (**A**) Variations in the relative abundance of the colon microbiota at the phylum level. (**B**) Variations in the relative abundance of the colon microbiota at the genus level. Different letters indicate significant differences (*p* < 0.05). Data were analyzed using one-way analysis of variance, followed by Duncan’s test.

As shown in Figure 2A, the phyla Firmicutes and Bacteroidetes were highly abundant, while the remaining phyla were represented by <5% of all sequences. Furthermore, no significant differences were observed at the phylum level among the three dietary treatment groups. *Prevotella* was the most abundant known genus, represented by 18.8% of all sequences. The abundance of *Prevotella* was higher (*p* = 0.72) in the control group (21.7%) than in the DON (17.5%) and ZEN (17.7%) groups (Figure 2B). The abundance of *Lactobacillus*, which was represented by 3.8% of the total sequences, was significantly different (*p* = 0.002) among the groups. Interestingly, *Lactobacillus* was significantly (*p* = 0.002) more abundant in the DON group (7.6%), compared to 2.7% and 0.2% in the ZEN and control groups, respectively. The abundance of *Megasphaera*, which was represented by 2.1% of all sequences, was significantly higher (*p* = 0.01) in the ZEN group (3.0%) than in the control group (1.7%), but no significant difference was observed between the DON (1.6%) and control groups. The abundance of *Faecalibacterium*, which was represented by 1.2% of the total sequences, was also significantly different (*p* = 0.045) among the three dietary treatment groups; compared with that in the control group (1.3%), the abundance was lower (0.7%) in the DON group, and slightly higher (1.5%) in the ZEN group. The abundance of the unclassified family Clostridiaceae was significantly different (*p* = 0.05) among the three dietary treatment groups, being much lower in the DON group (0.6%) and moderately lower in the ZEN group (1.2%) than that in the control group (2.1%).

Approximately 60% of the Firmicutes sequences could not be classified at the genus level. The following five unclassified groups were dominant: Ruminococcaceae (14.4%), Clostridiales (8.5%), Lachnospiraceae (3.8%), Veillonellaceae (2.7%), and Clostridiaceae (1.3%). Similar results have been reported for the feces of cattle fed different diets [23]; however, in the present study, none of the unclassified groups showed significant differences (*p* > 0.05) among the three dietary treatments. Approximately 32% of Bacteroidetes could not be classified at the genus level either, including S24-7, Bacteroidales, and Paraprevotellaceae, nor did these taxa show any differences (*p* > 0.05) among the dietary treatments.

### 2.3. Analysis of Diversity

The diversity of the pig colon microbiota was compared among the control and two mycotoxin-treated groups. We analyzed the species-level OTUs at a cutoff of 0.03 from all the samples to evaluate the diversity of the colon microbiota from the three dietary groups, and identified a total of 48,346 OTUs from all the samples. Nine of the 48,346 OTUs were represented by ≥0.1% of all sequences in at least one dietary group, but three of the nine OTUs could not be classified into known genera (Table 2).

OTU denovo31941, classified into Ruminococcaceae, was the most dominant among the nine OTUs in the collective data, and its abundance was the highest (*p* = 0.025) in the ZEN group and the lowest (*p* = 0.025) in the DON group (Table 2). The second most dominant OTU was that representing the genus *Megasphaera* (denovo92866), and it showed the highest abundance (*p* = 0.006) in the DON group and the lowest abundance (*p* = 0.006) in the control group. The next most abundant OTU was that representing the genus *Lactobacillus* (denovo28392), which was the most abundant (*p* = 0.006) in the DON group, moderately abundant (*p* = 0.006) in the ZEN group, and of the lowest abundance (*p* = 0.006) in the control group. Moreover, another OTU, also belonging to *Lactobacillus* (denovo218634), was highly, moderately, and lowly abundant (*p* = 0.049) in the DON, ZEN, and control groups, respectively. The abundances of three additional OTUs, representing Clostridiaceae (denovo231303; *p* = 0.019), *Bulleidia* (denovo254063; *p* = 0.011), and Clostridiales (denovo63294; *p* = 0.005), were much lower in the DON and ZEN groups than in the control group. An OTU of the genus *Prevotella* (denovo47686) was highly abundant in the ZEN group, moderately abundant in the DON group, and of the lowest abundance in the control group (*p* = 0.001). One OTU, classified as *Faecalibacterium*, was highly abundant in the ZEN group, and of relatively low abundance in the DON group compared with that in the control group (*p* = 0.001).

## 3. Discussion

Until now, few studies have been conducted to investigate the effects of the DON and ZEN mycotoxins on the pig gastrointestinal microbiota; in particular, the effects of ZEN on the gastrointestinal microbiota are largely unknown. Previous studies have examined the effects of naturally mycotoxin-contaminated feeds on the gut microbiota, but no reports are available on the microbial composition of the colonic contents of pigs fed commercially purified DON and ZEN [20,24]. Therefore, we analyzed the colon contents of pigs fed commercially purified DON and ZEN, to compare microbial abundances in the control, DON, and ZEN dietary groups, for the first time.

The animal digestive tract is suitable for microbial mass reproduction. Usually, various bacterial species are preferentially localized in different regions of the gastrointestinal tract, and not all regions are heavily colonized. According to Jia et al. [25], microbial communities in the small and large intestines play an important role in managing the host’s health, including the energy intake from food, immune system function, generation of important metabolites, and the response to gastrointestinal diseases. Swanson et al. [26] have reported the function of fecal and intestinal microbiota in the metabolism of trichothecenes, which was studied by anaerobically incubating microbial suspensions from the feces of swine, cattle, horses, dogs, chickens, and rats with diacetoxyscirpenol (DAS). The microbiota from pigs, cattle, and rats fully transformed DAS, primarily to de-epoxy monoacetoxyscirpenol and de-epoxy scirpentriol. This research clearly indicated that the function of intestinal microbiota involves more than just binding DAS. A study by McCormick [27] has reported anaerobic bacterial degradation of trichothecenes in animal systems. The toxic impact of trichothecenes on animals was mitigated by intestinal or rumen bacteria that could detoxify the compounds. Kabak and Dobson [28,29] have also discussed in their reviews the role of microorganisms in detoxification.

In the present study, Firmicutes (57.3%) and Bacteroidetes (35.0%) were the most abundant groups of bacteria in the colon contents from the DON, ZEN, and control groups at the phylum level, but their levels were not significantly different between the control and treatment groups. By contrast, abundances of Firmicutes and Bacteroidetes were significantly different in the ileum, cecum, and colon of growing pigs fed naturally DON-contaminated wheat [20]. Feeding a diet with a 100 µg/kg body weight dose of DON for 4 weeks by oral gavage increased the abundances of *Bacteroides* and *Prevotella* in rat intestines [30]. In another study, compared with the control group, the DON-treated mouse group showed significantly lower species abundances of Bacteroidetes and higher abundances of Proteobacteria [31]. According to Scaldaferri et al. [32], the abundance of Bacteroidetes significantly decreased as a result of intestinal ulceration or inflammation in humans. We speculated that these inconsistencies were due to variations in the type and age of animals, dietary concentrations of DON and ZEN, conditions of toxins (natural contamination or a purified powder), the duration of treatment, weather conditions, and the diet composition.

At the genus level, *Lactobacillus* was significantly more abundant in the DON and ZEN groups, particularly in the DON group, than in the control. Similar to our findings, *Lactobacillus* was the most abundant in the ileum, cecum, and colon of growing pigs fed a diet naturally contaminated with DON [20]. Some studies have already proven the many physiological effects of *Lactobacillus* on the host, including microbial interference, antimicrobial effects, nutritional supplementation, a decrease in the serum cholesterol, antitumor effects, a decrease in the necessary antibiotic treatments, and immunomodulatory effects, among others [20,33,34]. Investigators have previously found that the pig small intestine is commonly dominated by *Lactobacillus*, and generally, the bacterial density increases quickly as the animal grows [35,36]. *Lactobacillus* is an important natural constituent of the intestinal microbiota in healthy animals and humans; in fact, bacteria of this genus have been shown in in vitro and in vivo studies to bind various mycotoxins [37,38]. *Lactobacillus* spp. are also able to rapidly form a complex bacterial community, which protects the host from infections by pathogenic bacteria [39]. *Lactobacillus* can successfully remove trichothecenes, such as DON, fusarenon, and DAS from liquid media [40]. *Lactobacillus* species are potential probiotics, shown to participate in the removal of ZEN in an in vitro study [41]. El-Nezami and his team [42] have investigated the binding affinity interactions of ZEN and its derivative α-zearalenol with two food-grade strains of *Lactobacillus*. *Lactobacillus* spp. can produce lactic acid as a byproduct of carbohydrate fermentation, and have been reported to extracellularly bind ZEN, DON, and aflatoxins [42,43,44]. Cell wall polysaccharides and peptidoglycans are two key elements accountable for the binding of mutagens to *Lactobacillus* cells [45]. Consequently, the high proportion of *Lactobacillus*, detected in the current study, might have resulted from a competitive advantage related to the capability of *Lactobacillus* to metabolize DON and ZEN. Many investigators have examined the interactions between bacteria and trichothecenes and revealed that those can occur through metabolic degradation and the conversion of trichothecenes into considerably less toxic forms [46,47,48]. The effects of DON on the intestinal microbial abundance and diversity in mice have also been explored using an Illumina MiSeq high-throughput sequencing method [31]. Robert et al. [49] have demonstrated that the mucus and microbiota are important targets for dietary mycotoxins, especially DON. Using in vitro and ex vivo models, Pinton et al. [50] demonstrated that the inhibitory effect of DON on mucus secretion by human and porcine goblet cells relies on its ability to suppress the expression of the resistin-like molecule beta (RELM-beta). Generally, the presence of the gastrointestinal mucus can lower the capacity of probiotics to bind mycotoxins because the mucus may interfere with the adsorption of a mycotoxin to the bacterial cell wall of the probiotic. However, in the current study, the regular administration of the probiotic *Lactobacillus* during the dietary treatment period may have reduced the effect of the mucus. Consequently, it could be hypothesized that, as the number of colony-forming units increased, the effect of the mucus on the adsorption of DON and ZEN by the *Lactobacillus* cell wall was reduced. Previously, similar results have been reported by Gratz et al. [51] who used the probiotic *Lactobacillus* in aflatoxin B1-fed rats.

In the present study, the abundances of *Faecalibacterium* and the family Clostridiaceae were significantly lower in the DON and ZEN groups than in the control group. However, the relative abundances of these two bacterial taxa differed between the DON and ZEN dietary groups. This difference might have been caused by the use of DON and ZEN at different doses, which led to different effects on these two dominant bacterial taxa. These results suggest that the growth of these bacteria was inhibited by the highly toxic DON, and slightly inhibited by the low-toxic ZEN. Similarly, the abundance of *Clostridium* significantly decreased in the ileum and colon of DON-treated pigs, but increased in the colon of pigs fed DON-contaminated wheat in combination with *Clostridium* sp. WJ06, which is used as a detoxicant [20]. According to a study by Rotter et al. [52], before mycotoxin-contaminated food enters the small intestine of poultry and ruminants, DON comes into contact with high concentrations of microbes, which can change DON to de-epoxy-deoxynivalenol (DOM-1). These results suggest that DON may have induced intestinal lesions by destroying the integrity of the intestinal barrier in pigs, consistent with the data from previous studies on DON-fed pigs [20,53]. In the current study, bacteria of the genus *Megasphaera* were highly abundant in the ZEN and DON treatment groups, with no significant difference between the dietary groups. This genus is an important member of the gut microbiota, and exerts useful effects on the host [54]. Shetty et al. [55] have reported that *Megasphaera* plays a key role in the complex gut environment and performs the complete metabolic functions of the human gut microbiome. We assumed that because of the low concentration of ZEN, *Megasphaera* may have developed resistance to the low-toxic condition and, therefore, was abundant in the ZEN dietary group. Currently, there has been no published research on the microbial effects in ZEN-fed pigs. The absorption rates of DON and ZEN show substantial variance among animals; pigs show the highest absorption rate (82%), followed by chickens (19%), sheep (9.9%), and cows (1%) [56,57]. Frey et al. [58] have demonstrated that differences in absorption are mostly associated with the distribution of microorganisms before or after the small intestine. In particular, in monogastric animals and humans, bacteria are absent in the front portion of the small intestine; thus, compared to ruminants and poultry, pigs and humans are highly sensitive to DON [59,60]. Waché et al. [2] have revealed that DON-contaminated feed could increase the abundance of anaerobic bacteria in pigs, while decreasing the number of anaerobic sulfite-reducing bacteria. Based on the above observations, we speculated that the colon microbial structure and composition were mainly affected by the DON and ZEN doses, animal type, age, and health, as well as by environmental conditions and the duration of treatment.

To date, no OTU information has been available for intestinal or fecal samples from DON- or ZEN-treated pigs. In the present study, nine OTUs were identified whose abundances were significantly different among the three dietary treatment groups. Among these OTUs, two represented *Lactobacillus* and one each represented *Prevotella*, *Megasphaera*, *Bulleidia*, *Faecalibacterium*, unclassified Ruminococcaceae, unclassified Clostridiales, and unclassified Clostridiaceae. Similarly, except for *Megasphaera* and *Bulleidia,* the remaining seven OTU species were found in the colon contents of cattle fed different diets [23]. Species related to these nine main OTUs may have considerably contributed to the colon bacterial ecosystem, irrespective of the dietary treatment group. In particular, two OTUs representing *Lactobacillus* and *Megasphaera* were highly abundant in the DON group, and three OTUs, including *Prevotella, Faecalibacterium*, and Ruminococcaceae, were dominant in the ZEN group. Species associated with these OTUs may have played a major role in the metabolism of DON and ZEN. In particular, the *Lactobacillus* species corresponding to the two OTUs were shown to be highly abundant in the DON and ZEN groups, compared with their abundances in the control, and might have bound the DON and ZEN mycotoxins and detoxified them, protecting the pig immune system. For further clarification, isolation and characterization of *Lactobacillus* species will need to be performed to reveal their function in the pig gastrointestinal microbial community.

In summary, our results showed that the abundances of microbiota components were significantly different in the pig colon contents from the control, DON, and ZEN groups; in particular, the DON group showed the highest abundance of *Lactobacillus* among the treatment groups, possibly indicating a major role of *Lactobacillus* in detoxification. Based on the El-Nezami et al. [42] data, we believe that our results are due to the physical phenomenon of the binding of the DON and ZEN toxins to *Lactobacillus* via weak non-covalent bond interactions, such as those related to hydrophobic pockets on the bacterial surface. Consistent with this phenomenon, the *Lactobacillus* abundance was higher in the DON- and ZEN-treated groups than in the control group. There may be numerous tolerant, adaptable, and inducible members of the microbiota in the pig gastrointestinal tract following exposure to particular doses of DON and ZEN, but this possibility needs to be examined further. Additional studies are needed to investigate the chemical nature of mycotoxin-binding sites of *Lactobacillus*, and the types of chemical interactions involved in the binding mechanism. These future studies may also clarify the potential relationships between DON and ZEN, and the gut microbiota, facilitating the development of effective therapeutic methods to control DON- and ZEN-induced diseases.

## 4. Materials and Methods

### 4.1. Ethics Statement

The protocols for the animal experimental procedures were reviewed and approved by the Institutional Animal Care and Use Committee of the National Institute of Animal Science, South Korea (No. 2015-147, 29 May 2015).

### 4.2. Animals

The current study was carried out with 14 castrated 8-week-old male piglets (Landrace × Yorkshire = Large White Landrace; ~19 kg) obtained from a commercial pig farm. All piglets, which originated from different litters, were randomly selected. Each piglet was housed in a separate pen (2.1 m × 1.4 m) with free access to water from drinking nipples, and they were fed individually. The piglets were allowed to acclimate to their new housing conditions for 1 week at 25 ± 1 °C, and were subsequently allocated to three dietary groups, the control (*n* = 4), DON (*n* = 5), and ZEN (*n* = 5) groups, with approximately equal body weights. Piglets in the control group were supplied a standard diet (Table 3) to meet their nutritional requirements [61], and those in the treatment groups were supplied the control diet with added DON or ZEN. Commercially available purified DON and ZEN powders (Biomin Singapore Pte. Ltd., Singapore) were properly mixed into the feed at 8 mg/kg and 0.8 mg/kg, respectively. During the entire 4-week experimental period, the control and DON- and ZEN-contaminated diets, as well as water, were supplied to the pigs ad libitum. Ethical guidelines for animal protection rights were observed.

### 4.3. Mycotoxin Analysis

The DON and ZEN quantities in the DON- and ZEN-supplemented corn feed were examined by ultra-performance liquid chromatography (UPLC). A homogenized DON-contaminated grain sample (1 g) was extracted with 20 mL of distilled water by shaking for 30 min. A ZEN-contaminated corn sample (1 g) was mixed with 0.5 g of NaCl and 20 mL of acetonitrile (ACN), and then shaken for 1 h. After filtering the extracts through Whatman No. 1 paper, 5 mL of the DON-contaminated filtrate was diluted in 20 mL of phosphate-buffered saline (PBS), and 5 mL of the ZEN-mixed grain filtrate was diluted in 20 mL of a 1% Tween 20 solution. The extracted DON- and ZEN-mixed samples were loaded separately onto immunoaffinity chromatography columns. The DON column was allowed to dry, then washed with 10 mL each of PBS and distilled water, and eluted with 0.5 mL of methanol (MeOH) and 1.5 mL of ACN. In the case of ZEN, the column was washed with 10 mL of distilled water and eluted with 1.5 mL of MeOH. The eluates were dried under N_2_ gas, and 10 µL of each sample was injected into a UPLC instrument (Acquity UPLC^®^ H Class; Waters, Milford, MA, USA). The mobile phase used to separate DON and ZEN consisted of water/ACN/MeOH (90:5:5 for DON and 43:35:22 for ZEN). The samples were separated isocratically at a flow rate of 0.3 mL/min. Photodiode array and fluorescence detectors were used to detect DON and ZEN, respectively. Waters Acquity UPLC^®^ BEH C18 columns (2.1 × 100 mm, 1.7 μm particle size) were used for both DON and ZEN toxin analyses. The complete details of the mass spectrometry system running method, the excitation and emission wavelengths, and the limits of detection and quantification, were as described by Reddy et al. [7]. We found that the amounts of DON and ZEN in the mixed corn feed were 7.38 and 0.67 mg/kg, respectively; these values were close to the original concentrations. No DON and ZEN contamination was found in the control feed.

### 4.4. Sampling and Processing

A total of 14 male piglets were used to examine the colon microbiota composition in the standard diet and DON and ZEN mycotoxin-contaminated feed groups. After 4 weeks of treatment, all pigs were euthanized by an overdose of the anesthetic pentobarbital. After cardiac arrest, the colon contents of all pigs were aseptically collected directly into sample containers, rapidly frozen in liquid nitrogen, and then stored at −80 °C for further analysis.

### 4.5. DNA Extraction and 16S rRNA Gene Sequencing

Total community DNA was extracted from the 14 samples of colon contents using the repeated bead beating plus column method [62], and stored at −20 °C before sequencing. From each DNA sample, 14 amplicon libraries were generated using the 341F (5′-CCTACGGGNGGCWGCAG-3′) and 805R (5′-GACTACHVGGGTATCTAATCC-3′) primers, which produce approximately 450 bp products. The resultant 14 amplicon libraries were sequenced using a 2 × 300 bp paired-end protocol with the MiSeq platform (Illumina, San Diego, CA, USA) at the Macrogen sequencing facility (Macrogen, Inc., Seoul, Korea). Paired reads were assembled using the FLASH program [63]. The assembled sequences were demultiplexed using the default parameters and quality filtered using a Q20 minimum value with the QIIME software package 1.9.1 [64]. Taxa were identified using the Greengenes reference database [65], while OTUs were determined at 97% sequence similarity using the uclust program [66]. After 100,000 sequences were subsampled from each colon sample to normalize the number of OTUs, alpha diversity indices (number of OTUs, Chao1, PD_whole_tree distance, and Shannon diversity index) were determined.

### 4.6. Statistical Analysis

Taxa (or OTUs) representing, on average, ≥0.2% of the total sequences were regarded as major taxa (or OTUs), and used for statistical analysis. The mean proportion of each taxon or OTU in the total sequences was compared among the three diet groups using analysis of variance, followed by Duncan’s test, in the XLSTAT statistical software version 18.07 (Addinsoft, New York, NY, USA). A significant difference was considered at *p* < 0.05.

### 4.7. Nucleotide Sequence Accession Number

The 16S rRNA gene sequence data used in the current study are available from the EMBL European Nucleotide Archive under accession number PRJEB27663.

## Figures and Tables

**Table 1:** Diversity statistics of the reads in control and dietary deoxynivalenol (DON) and zearalenone (ZEN) treatment samples.

Sample Group	Sampling Type	No. of Sequences	No. of Observed OTUs ^1^	Chao1	Phylogenetic Diversity Whole Tree	Shannon	Sample Group
Control (*n* = 4)	Subsampled reads	100,000	16,961 ^a^	58,020 ^a^	791 ^a^	9.805 ^a^	0.990 ^a^
DON (*n* = 5)	Subsampled reads	100,000	16,003 ^a^	52,108 ^a,b^	772 ^a^	9.692 ^a^	0.991 ^a^
ZEN (*n* = 5)	Subsampled reads	100,000	14,565 ^a^	45,882 ^b^	696 ^a^	9.353 ^a^	0.989 ^a^

^1^ The number of operational taxonomic units (OTUs) was normalized by subsampling 100,000 sequences from each colon contents. Means among the three groups were compared using analysis of variance, followed by Duncan’s test. Values with different superscript letters in the same row are significantly different (*p* < 0.05).

**Table 2:** Relative abundances of significantly different operational taxonomic units (OTUs), calculated for the control, deoxynivalenol (DON), and zearalenone (ZEN) dietary groups.

OTU ID ^1^	Classification	Percentage of Total Sequences ^2^					
		Collective Data	Control	DON	ZEN	SEM	*p*-Value
denovo28392	*Lactobacillus*	1.03	0.01 ^b^	2.77 ^a^	0.29 ^a,b^	0.005	0.006
denovo31941	Unclassified Ruminococcaceae	2.32	2.39 ^a,b^	1.34 ^b^	3.23 ^a^	0.005	0.025
denovo47686	*Prevotella*	0.17	0.06 ^b^	0.14 ^a,b^	0.31 ^a^	0.001	0.001
denovo63294	Unclassified Clostridiales	0.21	0.32 ^a^	0.10 ^b^	0.21 ^a,b^	0.001	0.005
denovo92866	*Megasphaera*	1.13	0.63 ^b^	1.88 ^a^	0.89 ^b^	0.002	0.006
denovo218634	*Lactobacillus*	0.90	0.07 ^b^	1.57 ^a^	1.05 ^a,b^	0.004	0.049
denovo231303	Unclassified Clostridiaceae	0.39	0.77 ^a^	0.04 ^b^	0.34 ^a,b^	0.002	0.019
denovo254063	*Bulleidia*	0.18	0.29 ^a^	0.04 ^b^	0.22 ^a,b^	0.001	0.011
denovo274039	*Faecalibacterium*	0.39	0.45 ^a,b^	0.21 ^b^	0.52 ^a^	0.001	0.001

^1^ A total of 192,724 de novo OTUs were numbered in consecutive order. ^2^ Values represent the means. Values with different superscript letters in the same row are significantly different (*p* < 0.05).

**Table 3:** Ingredients and chemical composition of the piglet standard diet (as-fed basis).

Item	Control Diet
**Ingredients**	**(%)**
Ground corn	58.56
Soybean meal (46% crude protein)	14.00
Extruded soybean meal	12.00
Whey powder (12% crude protein)	7.00
Fish meal	3.45
Soybean oil	1.60
L-Lysine-HCl (78%)	0.43
DL-Methionine (99%)	0.14
L-Threonine (99%)	0.12
Calcium hydrophosphate	1.08
Limestone	0.60
Choline chloride (50%)	0.20
Sodium chloride	0.32
Vitamin–trace mineral premix ^1^	0.50
**Calculated nutrients**	**(%)**
Metabolizable energy (kcal/kg)	3444
Crude fiber	2.29
Crude protein	20.78
Crude fat	3.44
Ash	4.35
Lysine	1.47
Methionine	0.49
Calcium	0.75
Phosphorus	0.45

^1^ The following quantities (per kilogram of complete diet) were provided: vitamin A, 11,000 IU; vitamin D3, 1500 IU; vitamin E, 44.1 IU; vitamin K3, 4.0 mg; vitamin B1, 1.4 mg; vitamin B2, 5.22 mg; vitamin B5, 20.0 mg; vitamin B12, 0.01 mg; niacin, 26.0 mg; pantothenic acid, 14 mg; folic acid, 0.8 mg; biotin, 44 mg; Fe, 100.0 mg (as iron sulfate); Cu, 16.50 mg (as copper sulfate); Zn, 90.0 mg (as zinc sulfate); Mn, 35.0 mg (as manganese sulfate); I, 0.30 mg (as calcium iodate).

## Supplementary Materials

The following is available online at http://www.mdpi.com/2072-6651/10/9/347/s1, Table S1: Relative abundance of taxa in the control and DON and ZEN mycotoxin dietary treatment groups.
