# Supplementary material for: Colon Microbiome of Pigs Fed Diet Contaminated with Commercial Purified Deoxynivalenol and Zearalenone
Source: Toxins (Basel). 2018 Aug 29;10(9):347. doi: 10.3390/toxins10090347 (PMC6162637; doi:10.3390/toxins10090347)
Supplement: Supplementary file 1 [file toxins-10-00347-s001.pdf]

Supplementary Materials: Colon Microbiome of Pigs Fed Diet Contaminated with Commercial Purified Deoxynivalenol and Zearalenone

Kondreddy Eswar Reddy, Jin Young Jeong, Jaeyong Song, Yookyung Lee, Hyun-Jeong Lee, Dong-Wook Kim, Hyun Jung Jung, Ki Hyun Kim, Minji Kim, Young Kyoon Oh, Sung Dae Lee and Minseok Kim

Table S1. Relative abundance of taxa in the control and DON and ZEN mycotoxin dietary treatment groups.

Classification	Percentage of Total Sequences				SEM	*P*-value
	Collective Data	Control	DON	ZEN		
**Firmicutes**	57.3	55.5	57.3	58.6	0.030	0.81
Lactobacillus	3.8	0.2 [b]	7.6 [a]	2.7 [a,b]	0.010	0.002
Dialister	2.3	2.8	1.8	2.5	0.010	0.54
Megasphaera	2.1	1.7 [b]	1.6 [b]	3.0 [a]	0.004	0.01
Phascolarctobacterium	1.6	1.7	1.7	1.5	0.003	0.91
Ruminococcus	1.4	1.5	1.3	1.3	0.002	0.54
Faecalibacterium	1.2	1.3 [a,b]	0.7 [b]	1.5 [a]	0.002	0.045
Oscillospira	1.1	1.0	1.2	1.1	0.001	0.45
Lachnospira	1.0	1.4	0.8	0.7	0.002	0.09
Bulleidia	0.9	1.2	0.6	1.0	0.002	0.14
Coprococcus	0.8	1.1	0.6	0.8	0.001	0.10
Blautia	0.7	0.7	0.7	0.8	0.002	0.94
Shuttleworthia	0.7	1.3	0.8	0.2	0.005	0.39
p-75-a5	0.5	0.5	0.3	0.8	0.003	0.54
Dorea	0.5	0.3	0.7	0.5	0.001	0.18
Roseburia	0.5	0.8	0.3	0.5	0.002	0.24
Acidaminococcus	0.5	0.5	0.5	0.5	0.002	0.99
Clostridium	0.5	0.4	0.4	0.6	0.002	0.69
Catenibacterium	0.4	0.3	0.5	0.4	0.002	0.78
Anaerovibrio	0.4	0.3	0.4	0.4	0.001	0.93
Butyrivibrio	0.3	0.2	0.2	0.6	0.003	0.49
Eubacterium	0.3	0.3	0.3	0.4	0.001	0.86
Mitsuokella	0.3	0.3	0.2	0.3	0.001	0.85
Megamonas	0.3	0.0	0.0	0.7	0.005	0.44
RFN20	0.2	0.2	0.2	0.1	0.001	0.36
Epulopiscium	0.2	0.2	0.1	0.2	0.001	0.51
Unclassified Ruminococcaceae	14.4	14.4	15.4	15.7	0.015	0.83
Unclassified Clostridiales	8.5	9.1	7.5	8.9	0.001	0.32
Unclassified Lachnospiraceae	3.9	4.3	3.9	3.7	0.001	0.62
Unclassified Veillonellaceae	2.7	3.2	1.9	3.3	0.010	0.48
Unclassified Clostridiaceae	1.3	2.1 [a]	0.6[b]	1.2 [a,b]	0.003	0.05
Unclassified Christensenellaceae	0.5	0.5	0.5	0.3	0.002	0.61
Unclassified Erysipelotrichaceae	0.5	0.4	0.3	0.8	0.004	0.57
Unclassified Peptostreptococcaceae	0.2	0.2	0.3	0.2	0.001	0.25
**Bacteroidetes**	35.0	37.6	35.4	32.6	0.028	0.50
Prevotella	18.8	21.7	17.5	17.7	0.006	0.72
Bacteroides	2.3	2.8	1.8	2.5	0.008	0.68
CF231	1.2	1.0	1.5	1.1	0.003	0.54

Table S1. *Cont.*

**Classification**	**Percentage of Total Sequences**				**SEM**	***P*-value**
	**Collective Data**	**Control**	**DON**	**ZEN**		
Parabacteroides	0.8	0.8	1.1	0.6	0.003	0.53
Paludibacter	0.2	0.2	0.1	0.2	0.001	0.52
Unclassified S24-7	5.7	5.9	6.7	4.6	0.007	0.14
Unclassified Bacteroidales	2.8	2.6	3.4	2.4	0.006	0.51
Unclassified Paraprevotellaceae	2.5	2.2	2.4	2.9	0.002	0.26
**Proteobacteria**	3.2	2.6	2.3	4.5	0.011	0.30
Campylobacter	0.8	0.2	0.1	2.0	0.008	0.17
Desulfovibrio	0.5	0.4	0.4	0.6	0.001	0.48
Succinivibrio	0.3	0.5	0.1	0.3	0.002	0.47
**Spirochaetes**	1.7	1.8	2.0	1.3	0.009	0.86
Treponema	1.6	1.8	1.9	1.3	0.009	0.88
**Actinobacteria**	1.0	0.9	0.9	1.2	0.003	0.71
**Planctomycetes**	0.6	0.4	0.6	0.7	0.003	0.81
**Tenericutes**	0.5	0.4	0.5	0.4	0.001	0.79
**TM7**	0.5	0.4	0.5	0.4	0.001	0.72

[a, ab, b] Values with different superscript letters within the same row are significantly different ($P < 0.05$).
